# PD208 Optimal Scheme For Molecular Diagnosis Of *BRCA1/2* Gene Mutations In Patients With Breast Cancer In Poland - Cost-Utility Analysis

**DOI:** 10.1017/S0266462324004306

**Published:** 2025-01-07

**Authors:** Paweł Książek, Maurycy Muzyka, Agnieszka Nadzieja-Kozioł, Karolina Dziadek, Cezary Pruszko

## Abstract

**Introduction:**

Breast cancer is the most frequently diagnosed cancer among women in Poland. Molecular diagnosis of cancer, including identification of mutations in the BReast CAncer 1 and 2 (BRCA1/2) genes, is publicly funded in Poland. However, the diagnostic process is hampered by various billing models that use different testing materials and methods, so the diagnostic scheme is not considered optimal by physicians or patients.

**Methods:**

A cost-utility analysis was conducted to evaluate the cost effectiveness of modifying the molecular diagnosis protocol for breast cancer in Poland. The analysis compared the current diagnostic method with a new schema, which involves next-generation sequencing (NGS) for mutations in the BRCA1/2 genes for all patients with breast cancer. The model was developed using a comprehensive review of relevant literature, reimbursement data, and consultations with clinical experts.

**Results:**

If all patients with breast cancer undergo molecular diagnosis, the number of BRCA1/2 gene mutation carriers detected will increase from 213 to 971 per year. Despite the increase in costs to the public payer, compared with the existing scenario, the incremental health effect of the new scenario generated an incremental cost-effectiveness ratio of EUR37,274 per quality-adjusted life-year. This finding effectively disproves the claim that it is not cost effective to perform tests using the NGS technique in all patients with breast cancer.

**Conclusions:**

As a part of the cost-utility analysis it was shown that patients newly diagnosed with breast cancer should undergo molecular testing for mutations in the BRCA1/2 genes using NGS. This schema ensures patients’ access to appropriate targeted therapy. In addition, extended diagnosis of family members can prevent cancer in relatives who carry the mutation.

